# Poly[[μ_2_-aqua-aqua­(μ_3_-3,5-dinitro­salicylato)barium(II)] monohydrate]

**DOI:** 10.1107/S1600536808006338

**Published:** 2008-03-14

**Authors:** Wen-Dong Song, Run-Zhen Fan, Chang-Sheng Gu, Xiao-Min Hao

**Affiliations:** a College of Science, Guang Dong Ocean University, Zhanjiang 524088, People’s Republic of China

## Abstract

In the title coordination polymer, {[Ba(C_7_H_2_N_2_O_7_)(H_2_O)_2_]·H_2_O}_*n*_, the Ba^II^ atom is ten-coordinated by seven O atoms from four 3,5-dinitro­salicylatate ligands, two μ_2_-bridging aqua ligands and one water mol­ecule. The coordination mode is best described as a bicapped square-anti­prismatic geometry. The 3,5-dinitrosalicylatate ligands bridge three Ba atoms. Centrosymmetrically related dinuclear barium units, with a Ba⋯Ba separation of 4.767 (5) Å, form infinite chains, which are further self-assembled into a supra­molecular network through inter­molecular O—H⋯O hydrogen-bonding inter­actions between O atoms of 3,5-dinitro­salicylatate ligands and water mol­ecules.

## Related literature

For related literature, see: Song *et al.* (2007 ▶).
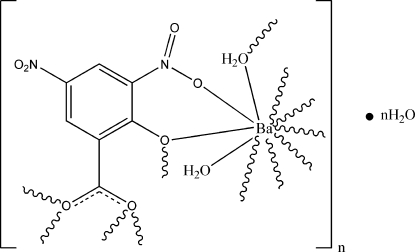


## Experimental

### 

#### Crystal data

[Ba(C_7_H_2_N_2_O_7_)(H_2_O)_2_]·H_2_O
*M* *_r_* = 417.49
Monoclinic, 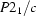

*a* = 11.9649 (6) Å
*b* = 4.1866 (2) Å
*c* = 26.121 (1) Å
β = 109.332 (3)°
*V* = 1234.7 (1) Å^3^
*Z* = 4
Mo *K*α radiation
μ = 3.27 mm^−1^
*T* = 296 (2) K
0.30 × 0.26 × 0.23 mm

#### Data collection

Bruker APEXII area-detector diffractometer
Absorption correction: multi-scan (*SADABS*; Sheldrick, 1996 ▶) *T*_min_ = 0.392, *T*_max_ = 0.471
8615 measured reflections
2374 independent reflections
2189 reflections with *I* > 2σ(*I*)
*R*_int_ = 0.041

#### Refinement

*R*[*F* ^2^ > 2σ(*F* ^2^)] = 0.026
*wR*(*F* ^2^) = 0.067
*S* = 1.05
2374 reflections
199 parameters
9 restraints
H atoms treated by a mixture of independent and constrained refinement
Δρ_max_ = 1.03 e Å^−3^
Δρ_min_ = −1.30 e Å^−3^

### 

Data collection: *APEX2* (Bruker, 2004 ▶); cell refinement: *SAINT* (Bruker, 2004 ▶); data reduction: *SAINT*; program(s) used to solve structure: *SHELXS97* (Sheldrick, 2008 ▶); program(s) used to refine structure: *SHELXL97* (Sheldrick, 2008 ▶); molecular graphics: *XP* in *SHELXTL* (Sheldrick, 2008 ▶); software used to prepare material for publication: *SHELXTL*.

## Supplementary Material

Crystal structure: contains datablocks I, global. DOI: 10.1107/S1600536808006338/im2052sup1.cif

Structure factors: contains datablocks I. DOI: 10.1107/S1600536808006338/im2052Isup2.hkl

Additional supplementary materials:  crystallographic information; 3D view; checkCIF report

## Figures and Tables

**Table 1:** Hydrogen-bond geometry (Å, °)

*D*—H⋯*A*	*D*—H	H⋯*A*	*D*⋯*A*	*D*—H⋯*A*
O3*W*—H6*W*⋯O7^i^	0.82 (3)	2.27 (3)	2.916 (4)	135 (4)
O3*W*—H5*W*⋯O5^ii^	0.82 (4)	2.60 (4)	2.985 (4)	110 (3)
O3*W*—H5*W*⋯O2*W*^iii^	0.82 (4)	2.04 (3)	2.755 (4)	145 (4)
O2*W*—H4*W*⋯N1^iv^	0.83 (3)	2.69 (4)	3.340 (4)	137 (4)
O2*W*—H4*W*⋯O4^iv^	0.83 (3)	2.55 (4)	3.080 (4)	123 (3)
O2*W*—H4*W*⋯O5^iv^	0.83 (3)	2.25 (3)	2.993 (4)	150 (5)
O2*W*—H3*W*⋯O3^v^	0.83 (3)	2.01 (2)	2.730 (4)	145 (4)
O1*W*—H1*W*⋯O3*W*^v^	0.83 (3)	1.991 (16)	2.798 (4)	164 (4)
O1*W*—H2*W*⋯O3*W*^vi^	0.83 (3)	1.90 (3)	2.725 (4)	171 (4)

Symmetry codes: (i) 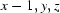; (ii) 
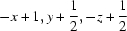
; (iii) 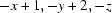; (iv) 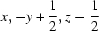; (v) 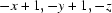; (vi) 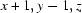.

**Fig. 1.**
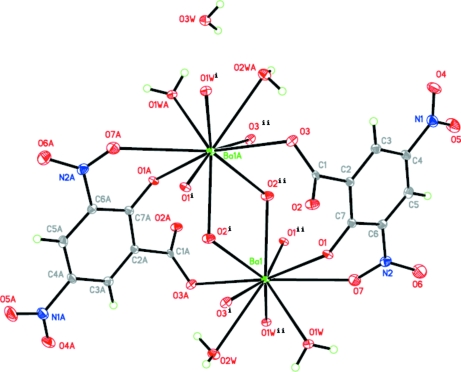
The structure of (I), showing the atomic numbering scheme. Non-H atoms are shown with 30% probability displacement ellipsoids.

**Fig. 2.**
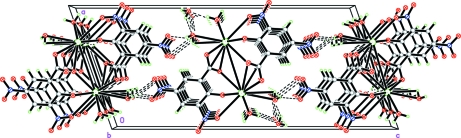
A packing view of the title compound. The intermolecluar hydrogen bonds are shown as dashed lines.

## supplementary crystallographic information

### Comment

In the structural investigation of 3,5-dinitrosalicylatato complexes, it has been found that the 3,5-dinitrosalicylatato moiety functions as a multidentate ligand (Song *et al.*, 2007) with versatile binding and coordination modes. In this paper, we report the crystal structure of the title compound, (I), a new Ba complex obtained by the reaction of 3,5-dinitrosalicylic acid and barium chloride in alkaline aqueous solution.

As illustrated in Figure 1, the Ba^II^ atom displays a bicapped square antiprismatic coordination environment, defined by seven O atoms from four 3,5-dinitrosalicylatato ligands, two µ_2_-bridging aqua ligands and one water molecule. The 3,5-dinitrosalicylatato ligands link barium ions to form infinite chains, which are further self-assembled into a supramolecular network through intermolecular O—H···O hydrogen bonding interactions (Table 1) involving the uncoordinating water molecules, coordinating water molecules as donors and O atoms of 3,5-dinitrosalicylatato ligands as acceptors (Fig. 2).

### Experimental

A mixture of barium chloride (1 mmol), 3,5-dinitrosalicylic acid (1 mmol), NaOH (1.5 mmol) and H_2_O (12 ml) was placed in a 23 ml Teflon reactor, which was heated to 433 K for three days and then cooled to room temperature at a rate of 10 K h^-1^. The obtained crystals obtained were washed with water and dryed in air.

### Refinement

Carbon-bound H atoms were placed at calculated positions and were treated as riding on the parent C atoms with C—H = 0.93 Å, and with *U*_iso_(H) = 1.2 *U*_eq_(C). Water H atoms were tentatively located in difference Fourier maps and were refined with distance restraints of O–H = 0.84 Å and H···H = 1.39 Å, each within a standard deviation of 0.01 Å, and with *U*_iso_(H) = 1.5 *U*_eq_(O)

### Crystal data

**Table:**

[Ba(C_7_H_2_N_2_O_7_)(H_2_O)_2_]·H_2_O	*F*_000_ = 800
*M**_r_* = 417.49	*D*_x_ = 2.246 Mg m^−^^3^
Monoclinic, *P*2_1_/*c*	Mo *K*α radiation λ = 0.71073 Å
Hall symbol: -P 2ybc	Cell parameters from 5837 reflections
*a* = 11.9649 (6) Å	θ = 2.8–27.9º
*b* = 4.1866 (2) Å	µ = 3.27 mm^−^^1^
*c* = 26.121 (1) Å	*T* = 296 (2) K
β = 109.332 (3)º	Block, yellow
*V* = 1234.7 (1) Å^3^	0.30 × 0.26 × 0.23 mm
*Z* = 4	

### Data collection

**Table:**

Bruker APEXII area-detector diffractometer	2374 independent reflections
Radiation source: fine-focus sealed tube	2189 reflections with *I* > 2σ(*I*)
Monochromator: graphite	*R*_int_ = 0.041
*T* = 296(2) K	θ_max_ = 26.0º
φ and ω scans	θ_min_ = 1.7º
Absorption correction: multi-scan(SADABS; Sheldrick, 1996)	*h* = −14→14
*T*_min_ = 0.392, *T*_max_ = 0.472	*k* = −4→4
8615 measured reflections	*l* = −31→32

### Refinement

**Table:**

Refinement on *F*^2^	Secondary atom site location: difference Fourier map
Least-squares matrix: full	Hydrogen site location: inferred from neighbouring sites
*R*[*F*^2^ > 2σ(*F*^2^)] = 0.026	H atoms treated by a mixture of independent and constrained refinement
*wR*(*F*^2^) = 0.067	*w* = 1/[σ^2^(*F*_o_^2^) + (0.0339*P*)^2^ + 1.4739*P*] where *P* = (*F*_o_^2^ + 2*F*_c_^2^)/3
*S* = 1.05	(Δ/σ)_max_ = 0.001
2374 reflections	Δρ_max_ = 1.03 e Å^−^^3^
199 parameters	Δρ_min_ = −1.30 e Å^−^^3^
9 restraints	Extinction correction: none
Primary atom site location: structure-invariant direct methods	

### Special details

**Table:**

Geometry. All e.s.d.'s (except the e.s.d. in the dihedral angle between two l.s. planes) are estimated using the full covariance matrix. The cell e.s.d.'s are taken into account individually in the estimation of e.s.d.'s in distances, angles and torsion angles; correlations between e.s.d.'s in cell parameters are only used when they are defined by crystal symmetry. An approximate (isotropic) treatment of cell e.s.d.'s is used for estimating e.s.d.'s involving l.s. planes.
Refinement. Refinement of *F*^2^ against ALL reflections. The weighted *R*-factor *wR* and goodness of fit *S* are based on *F*^2^, conventional *R*-factors *R* are based on *F*, with *F* set to zero for negative *F*^2^. The threshold expression of *F*^2^ > σ(*F*^2^) is used only for calculating *R*-factors(gt) *etc*. and is not relevant to the choice of reflections for refinement. *R*-factors based on *F*^2^ are statistically about twice as large as those based on *F*, and *R*- factors based on ALL data will be even larger.

### Fractional atomic coordinates and isotropic or equivalent isotropic displacement parameters (Å^2^)

**Table:**

	*x*	*y*	*z*	*U*_iso_*/*U*_eq_	
Ba1	0.698106 (16)	0.59849 (4)	0.002749 (8)	0.01250 (10)	
O1	0.7177 (2)	0.1028 (5)	0.06998 (10)	0.0150 (5)	
O2	0.5320 (2)	−0.3574 (6)	0.05297 (11)	0.0211 (6)	
O3	0.4012 (2)	−0.0694 (6)	0.07602 (11)	0.0190 (6)	
O4	0.5934 (3)	0.0066 (8)	0.28282 (12)	0.0311 (7)	
O5	0.7491 (3)	0.3054 (8)	0.31157 (12)	0.0359 (7)	
O6	0.9954 (3)	0.3764 (8)	0.19294 (14)	0.0416 (9)	
O7	0.8915 (2)	0.5460 (7)	0.11347 (12)	0.0251 (6)	
N1	0.6755 (3)	0.1544 (8)	0.27501 (14)	0.0245 (7)	
N2	0.9007 (3)	0.4015 (7)	0.15565 (14)	0.0205 (7)	
C1	0.5067 (3)	−0.1530 (8)	0.08248 (14)	0.0116 (7)	
C2	0.6052 (3)	−0.0051 (9)	0.12815 (14)	0.0129 (7)	
C3	0.5950 (3)	0.0128 (9)	0.17862 (15)	0.0167 (7)	
H3	0.5270	−0.0629	0.1844	0.020*	
C4	0.6874 (3)	0.1461 (9)	0.22201 (15)	0.0178 (8)	
C5	0.7878 (3)	0.2691 (9)	0.21433 (15)	0.0191 (8)	
H5	0.8488	0.3552	0.2431	0.023*	
C6	0.7951 (3)	0.2603 (9)	0.16271 (14)	0.0158 (7)	
C7	0.7074 (3)	0.1200 (8)	0.11687 (15)	0.0146 (8)	
O1W	0.8608 (2)	0.1295 (6)	0.00092 (12)	0.0205 (6)	
H2W	0.922 (2)	0.101 (10)	0.0272 (9)	0.031*	
H1W	0.881 (3)	0.137 (10)	−0.0265 (9)	0.031*	
O2W	0.7483 (2)	0.6467 (6)	−0.09980 (12)	0.0223 (6)	
H3W	0.688 (2)	0.758 (8)	−0.1068 (17)	0.033*	
H4W	0.732 (3)	0.484 (6)	−0.1189 (16)	0.033*	
O3W	0.0565 (2)	0.9715 (8)	0.08622 (12)	0.0255 (6)	
H5W	0.110 (3)	1.093 (8)	0.1033 (15)	0.038*	
H6W	0.047 (4)	0.837 (8)	0.1074 (13)	0.038*	

### Atomic displacement parameters (Å^2^)

**Table:**

	*U*^11^	*U*^22^	*U*^33^	*U*^12^	*U*^13^	*U*^23^
Ba1	0.01402 (13)	0.01055 (14)	0.01455 (15)	−0.00009 (7)	0.00690 (10)	−0.00038 (8)
O1	0.0191 (13)	0.0156 (14)	0.0133 (13)	−0.0010 (9)	0.0091 (11)	−0.0007 (10)
O2	0.0199 (13)	0.0222 (15)	0.0235 (15)	−0.0031 (10)	0.0105 (12)	−0.0089 (11)
O3	0.0142 (12)	0.0185 (15)	0.0238 (15)	0.0005 (10)	0.0055 (11)	−0.0015 (11)
O4	0.0331 (16)	0.0413 (17)	0.0244 (16)	−0.0037 (14)	0.0167 (13)	0.0019 (15)
O5	0.0411 (18)	0.0464 (19)	0.0186 (16)	−0.0102 (15)	0.0077 (14)	−0.0100 (15)
O6	0.0227 (16)	0.066 (3)	0.033 (2)	−0.0138 (14)	0.0056 (15)	0.0021 (16)
O7	0.0248 (14)	0.0226 (15)	0.0300 (17)	−0.0044 (11)	0.0117 (12)	0.0052 (13)
N1	0.0296 (18)	0.0263 (18)	0.0184 (18)	0.0037 (14)	0.0089 (15)	−0.0003 (14)
N2	0.0151 (15)	0.0223 (19)	0.0230 (19)	−0.0046 (12)	0.0049 (14)	−0.0040 (14)
C1	0.0132 (16)	0.0130 (18)	0.0082 (17)	−0.0010 (13)	0.0030 (14)	0.0023 (13)
C2	0.0163 (16)	0.0102 (17)	0.0128 (18)	0.0025 (14)	0.0056 (14)	0.0018 (14)
C3	0.0178 (17)	0.0153 (18)	0.0178 (19)	0.0006 (15)	0.0071 (15)	0.0015 (16)
C4	0.0214 (18)	0.021 (2)	0.0116 (18)	0.0022 (14)	0.0060 (15)	−0.0015 (15)
C5	0.0190 (17)	0.018 (2)	0.0166 (19)	−0.0003 (15)	0.0013 (15)	−0.0030 (16)
C6	0.0129 (16)	0.016 (2)	0.0181 (19)	−0.0012 (14)	0.0042 (14)	−0.0002 (15)
C7	0.0184 (18)	0.0121 (19)	0.0146 (19)	0.0044 (13)	0.0074 (15)	0.0040 (13)
O1W	0.0156 (13)	0.0291 (16)	0.0186 (15)	0.0036 (10)	0.0079 (11)	0.0005 (12)
O2W	0.0290 (15)	0.0188 (15)	0.0222 (15)	0.0010 (11)	0.0126 (13)	−0.0019 (11)
O3W	0.0206 (14)	0.0334 (17)	0.0235 (16)	−0.0020 (12)	0.0086 (12)	0.0046 (13)

### Geometric parameters (Å, °)

**Table:**

Ba1—O1	2.678 (2)	O5—N1	1.237 (4)
Ba1—O1^i^	2.706 (2)	O6—N2	1.230 (5)
Ba1—O2^i^	2.726 (3)	O7—N2	1.230 (4)
Ba1—O1W	2.777 (3)	N1—C4	1.438 (5)
Ba1—O3^ii^	2.813 (3)	N2—C6	1.462 (4)
Ba1—O2^iii^	2.840 (3)	C1—C2	1.505 (5)
Ba1—O2W	2.940 (3)	C1—Ba1^iii^	3.290 (3)
Ba1—O1W^i^	2.966 (3)	C2—C3	1.366 (5)
Ba1—O3^iii^	2.989 (3)	C2—C7	1.447 (5)
Ba1—O7	3.056 (3)	C3—C4	1.410 (5)
Ba1—C1^iii^	3.290 (3)	C3—H3	0.9300
Ba1—Ba1^i^	4.18660 (19)	C4—C5	1.382 (5)
Ba1—H3W	2.90 (5)	C5—C6	1.380 (5)
O1—C7	1.273 (4)	C5—H5	0.9300
O1—Ba1^iv^	2.706 (2)	C6—C7	1.431 (5)
O2—C1	1.254 (4)	O1W—Ba1^iv^	2.966 (3)
O2—Ba1^iv^	2.726 (3)	O1W—H2W	0.83 (4)
O2—Ba1^iii^	2.840 (3)	O1W—H1W	0.83 (4)
O3—C1	1.266 (4)	O2W—H3W	0.83 (4)
O3—Ba1^ii^	2.813 (3)	O2W—H4W	0.83 (4)
O3—Ba1^iii^	2.989 (3)	O3W—H5W	0.82 (4)
O4—N1	1.233 (4)	O3W—H6W	0.83 (4)
			
O1—Ba1—O1^i^	102.07 (8)	O7—Ba1—Ba1^i^	94.12 (5)
O1—Ba1—O2^i^	69.92 (8)	C1^iii^—Ba1—Ba1^i^	124.53 (6)
O1^i^—Ba1—O2^i^	63.59 (7)	O1—Ba1—H3W	142.3 (6)
O1—Ba1—O1W	63.49 (7)	O1^i^—Ba1—H3W	115.3 (6)
O1^i^—Ba1—O1W	130.70 (8)	O2^i^—Ba1—H3W	131.3 (3)
O2^i^—Ba1—O1W	133.10 (8)	O1W—Ba1—H3W	86.9 (3)
O1—Ba1—O3^ii^	161.23 (8)	O3^ii^—Ba1—H3W	41.2 (3)
O1^i^—Ba1—O3^ii^	81.53 (7)	O2^iii^—Ba1—H3W	81.9 (7)
O2^i^—Ba1—O3^ii^	96.08 (8)	O2W—Ba1—H3W	16.3 (6)
O1W—Ba1—O3^ii^	127.72 (8)	O1W^i^—Ba1—H3W	68.0 (7)
O1—Ba1—O2^iii^	85.43 (8)	O3^iii^—Ba1—H3W	67.3 (7)
O1^i^—Ba1—O2^iii^	118.10 (7)	O7—Ba1—H3W	136.2 (6)
O2^i^—Ba1—O2^iii^	62.17 (9)	C1^iii^—Ba1—H3W	71.6 (7)
O1W—Ba1—O2^iii^	107.83 (7)	Ba1^i^—Ba1—H3W	76.7 (6)
O3^ii^—Ba1—O2^iii^	76.78 (8)	C7—O1—Ba1	124.9 (2)
O1—Ba1—O2W	130.60 (7)	C7—O1—Ba1^iv^	130.8 (2)
O1^i^—Ba1—O2W	122.52 (7)	Ba1—O1—Ba1^iv^	102.07 (8)
O2^i^—Ba1—O2W	146.64 (8)	C1—O2—Ba1^iv^	134.8 (2)
O1W—Ba1—O2W	71.15 (8)	C1—O2—Ba1^iii^	99.6 (2)
O3^ii^—Ba1—O2W	56.60 (7)	Ba1^iv^—O2—Ba1^iii^	117.83 (9)
O2^iii^—Ba1—O2W	90.76 (8)	C1—O3—Ba1^ii^	116.9 (2)
O1—Ba1—O1W^i^	132.52 (7)	C1—O3—Ba1^iii^	92.2 (2)
O1^i^—Ba1—O1W^i^	60.61 (7)	Ba1^ii^—O3—Ba1^iii^	92.33 (8)
O2^i^—Ba1—O1W^i^	122.95 (7)	N2—O7—Ba1	134.3 (2)
O1W—Ba1—O1W^i^	93.55 (7)	O4—N1—O5	122.1 (3)
O3^ii^—Ba1—O1W^i^	65.38 (7)	O4—N1—C4	119.0 (3)
O2^iii^—Ba1—O1W^i^	142.04 (8)	O5—N1—C4	118.9 (3)
O2W—Ba1—O1W^i^	66.46 (8)	O7—N2—O6	122.5 (3)
O1—Ba1—O3^iii^	78.80 (7)	O7—N2—C6	119.2 (3)
O1^i^—Ba1—O3^iii^	162.60 (7)	O6—N2—C6	118.3 (3)
O2^i^—Ba1—O3^iii^	101.22 (7)	O2—C1—O3	122.7 (3)
O1W—Ba1—O3^iii^	65.50 (7)	O2—C1—C2	118.9 (3)
O3^ii^—Ba1—O3^iii^	92.33 (8)	O3—C1—C2	118.5 (3)
O2^iii^—Ba1—O3^iii^	44.50 (7)	O2—C1—Ba1^iii^	58.32 (18)
O2W—Ba1—O3^iii^	65.05 (7)	O3—C1—Ba1^iii^	65.18 (18)
O1W^i^—Ba1—O3^iii^	131.14 (7)	C2—C1—Ba1^iii^	169.1 (2)
O1—Ba1—O7	56.58 (7)	C3—C2—C7	121.9 (3)
O1^i^—Ba1—O7	64.28 (8)	C3—C2—C1	119.4 (3)
O2^i^—Ba1—O7	89.64 (8)	C7—C2—C1	118.7 (3)
O1W—Ba1—O7	69.50 (8)	C2—C3—C4	120.0 (3)
O3^ii^—Ba1—O7	138.31 (7)	C2—C3—H3	120.0
O2^iii^—Ba1—O7	139.60 (8)	C4—C3—H3	120.0
O2W—Ba1—O7	123.25 (7)	C5—C4—C3	121.3 (3)
O1W^i^—Ba1—O7	76.92 (7)	C5—C4—N1	119.9 (3)
O3^iii^—Ba1—O7	127.03 (7)	C3—C4—N1	118.8 (3)
O1—Ba1—C1^iii^	83.60 (8)	C6—C5—C4	118.1 (3)
O1^i^—Ba1—C1^iii^	140.04 (8)	C6—C5—H5	121.0
O2^i^—Ba1—C1^iii^	82.29 (8)	C4—C5—H5	121.0
O1W—Ba1—C1^iii^	87.53 (8)	C5—C6—C7	124.2 (3)
O3^ii^—Ba1—C1^iii^	82.12 (8)	C5—C6—N2	116.7 (3)
O2^iii^—Ba1—C1^iii^	22.07 (8)	C7—C6—N2	119.1 (3)
O2W—Ba1—C1^iii^	75.72 (8)	O1—C7—C6	123.4 (3)
O1W^i^—Ba1—C1^iii^	139.44 (8)	O1—C7—C2	122.2 (3)
O3^iii^—Ba1—C1^iii^	22.61 (8)	C6—C7—C2	114.4 (3)
O7—Ba1—C1^iii^	139.53 (8)	Ba1—O1W—Ba1^iv^	93.55 (7)
O1—Ba1—Ba1^i^	140.79 (5)	Ba1—O1W—H2W	121 (3)
O1^i^—Ba1—Ba1^i^	38.72 (5)	Ba1^iv^—O1W—H2W	107 (3)
O2^i^—Ba1—Ba1^i^	86.11 (5)	Ba1—O1W—H1W	113 (3)
O1W—Ba1—Ba1^i^	135.00 (5)	Ba1^iv^—O1W—H1W	115 (3)
O3^ii^—Ba1—Ba1^i^	45.50 (5)	H2W—O1W—H1W	106.4 (17)
O2^iii^—Ba1—Ba1^i^	110.82 (5)	Ba1—O2W—H3W	79 (3)
O2W—Ba1—Ba1^i^	86.06 (5)	Ba1—O2W—H4W	114 (4)
O1W^i^—Ba1—Ba1^i^	41.45 (5)	H3W—O2W—H4W	108 (4)
O3^iii^—Ba1—Ba1^i^	137.83 (5)	H5W—O3W—H6W	108 (4)

Symmetry codes: (i) *x*, *y*+1, *z*; (ii) −*x*+1, −*y*+1, −*z*; (iii) −*x*+1, −*y*, −*z*; (iv) *x*, *y*−1, *z*.

### Hydrogen-bond geometry (Å, °)

**Table:**

*D*—H···*A*	*D*—H	H···*A*	*D*···*A*	*D*—H···*A*
O3W—H6W···O7^v^	0.82 (3)	2.27 (3)	2.916 (4)	135 (4)
O3W—H5W···O5^vi^	0.82 (4)	2.60 (4)	2.985 (4)	110 (3)
O3W—H5W···O2W^vii^	0.82 (4)	2.04 (3)	2.755 (4)	145 (4)
O2W—H4W···N1^viii^	0.83 (3)	2.69 (4)	3.340 (4)	137 (4)
O2W—H4W···O4^viii^	0.83 (3)	2.55 (4)	3.080 (4)	123 (3)
O2W—H4W···O5^viii^	0.83 (3)	2.25 (3)	2.993 (4)	150 (5)
O2W—H3W···O3^ii^	0.83 (3)	2.01 (2)	2.730 (4)	145 (4)
O1W—H1W···O3W^ii^	0.83 (3)	1.991 (16)	2.798 (4)	164 (4)
O1W—H2W···O3W^ix^	0.83 (3)	1.90 (3)	2.725 (4)	171 (4)

Symmetry codes: (v) *x*−1, *y*, *z*; (vi) −*x*+1, *y*+1/2, −*z*+1/2; (vii) −*x*+1, −*y*+2, −*z*; (viii) *x*, −*y*+1/2, *z*−1/2; (ii) −*x*+1, −*y*+1, −*z*; (ix) *x*+1, *y*−1, *z*.
